# New strategies for characterizing genetic structure in wide-ranging, continuously distributed species: A Greater Sage-grouse case study

**DOI:** 10.1371/journal.pone.0274189

**Published:** 2022-09-13

**Authors:** Sara J. Oyler-McCance, Todd B. Cross, Jeffery R. Row, Michael K. Schwartz, Dave E. Naugle, Jennifer A. Fike, Kristopher Winiarski, Brad C. Fedy

**Affiliations:** 1 U. S. Geological Survey, Fort Collins Science Center, Fort Collins, Colorado, United States of America; 2 School of Environment, Resources and Sustainability, University of Waterloo, Waterloo, Ontario, Canada; 3 USDA Forest Service, National Genomics Center for Wildlife and Fish Conservation, Missoula, Montana, United States of America; 4 Wildlife Biology Program, W. A. Franke College of Forestry and Conservation, University of Montana, Missoula, Montana, United States of America; University of Arkansas Fayetteville, UNITED STATES

## Abstract

Characterizing genetic structure across a species’ range is relevant for management and conservation as it can be used to define population boundaries and quantify connectivity. Wide-ranging species residing in continuously distributed habitat pose substantial challenges for the characterization of genetic structure as many analytical methods used are less effective when isolation by distance is an underlying biological pattern. Here, we illustrate strategies for overcoming these challenges using a species of significant conservation concern, the Greater Sage-grouse (*Centrocercus urophasianus*), providing a new method to identify centers of genetic differentiation and combining multiple methods to help inform management and conservation strategies for this and other such species. Our objectives were to (1) describe large-scale patterns of population genetic structure and gene flow and (2) to characterize genetic subpopulation centers across the range of Greater Sage-grouse. Samples from 2,134 individuals were genotyped at 15 microsatellite loci. Using standard STRUCTURE and spatial principal components analyses, we found evidence for four or six areas of large-scale genetic differentiation and, following our novel method, 12 subpopulation centers of differentiation. Gene flow was greater, and differentiation reduced in areas of contiguous habitat (eastern Montana, most of Wyoming, much of Oregon, Nevada, and parts of Idaho). As expected, areas of fragmented habitat such as in Utah (with 6 subpopulation centers) exhibited the greatest genetic differentiation and lowest effective migration. The subpopulation centers defined here could be monitored to maintain genetic diversity and connectivity with other subpopulation centers. Many areas outside subpopulation centers are contact zones where different genetic groups converge and could be priorities for maintaining overall connectivity. Our novel method and process of leveraging multiple different analyses to find common genetic patterns provides a path forward to characterizing genetic structure in wide-ranging, continuously distributed species.

## Introduction

Many wildlife species are comprised of multiple spatially disjunct subgroups and the amount and juxtaposition of this genetic structure across the landscape is shaped by a species’ social behavior, their ability to disperse, and the spatial heterogeneity of the landscape [1, 2]. Movement and connectivity among genetic groups is important for population demography and persistence as well as the maintenance of genetic diversity [3–8]. With increasing fragmentation in landscapes due to human activity, populations can become more differentiated and isolated, and those that are small can ultimately lose genetic diversity due to genetic drift and may face problems with inbreeding. Characterizing genetic structure within a species is highly relevant for management and conservation as it can be used to define population boundaries and management units as well as assess connectivity, all of which can help prioritize where and how to invest scarce resources for conservation [9, 10].

Improvements in molecular technology and laboratory methods over the last decade have facilitated the collection and analysis of large-scale genetic data sets [9, 11–13] enabling comprehensive research efforts for wide-ranging species. An array of analytical tools exists to examine population genetic structure in wild populations including both population and individual-based methods [14] as well as those that include spatial information and those that do not [15]. Such methods are useful for delineating genetic groups yet may be less useful for identifying areas that are central to maintaining genetic variation and connectivity across large landscapes [16]. In addition, many of these methods are less effective when used in situations where habitat is continuous and isolation by distance (IBD) is an underlying biological function [17–20].

**Fig 1:**
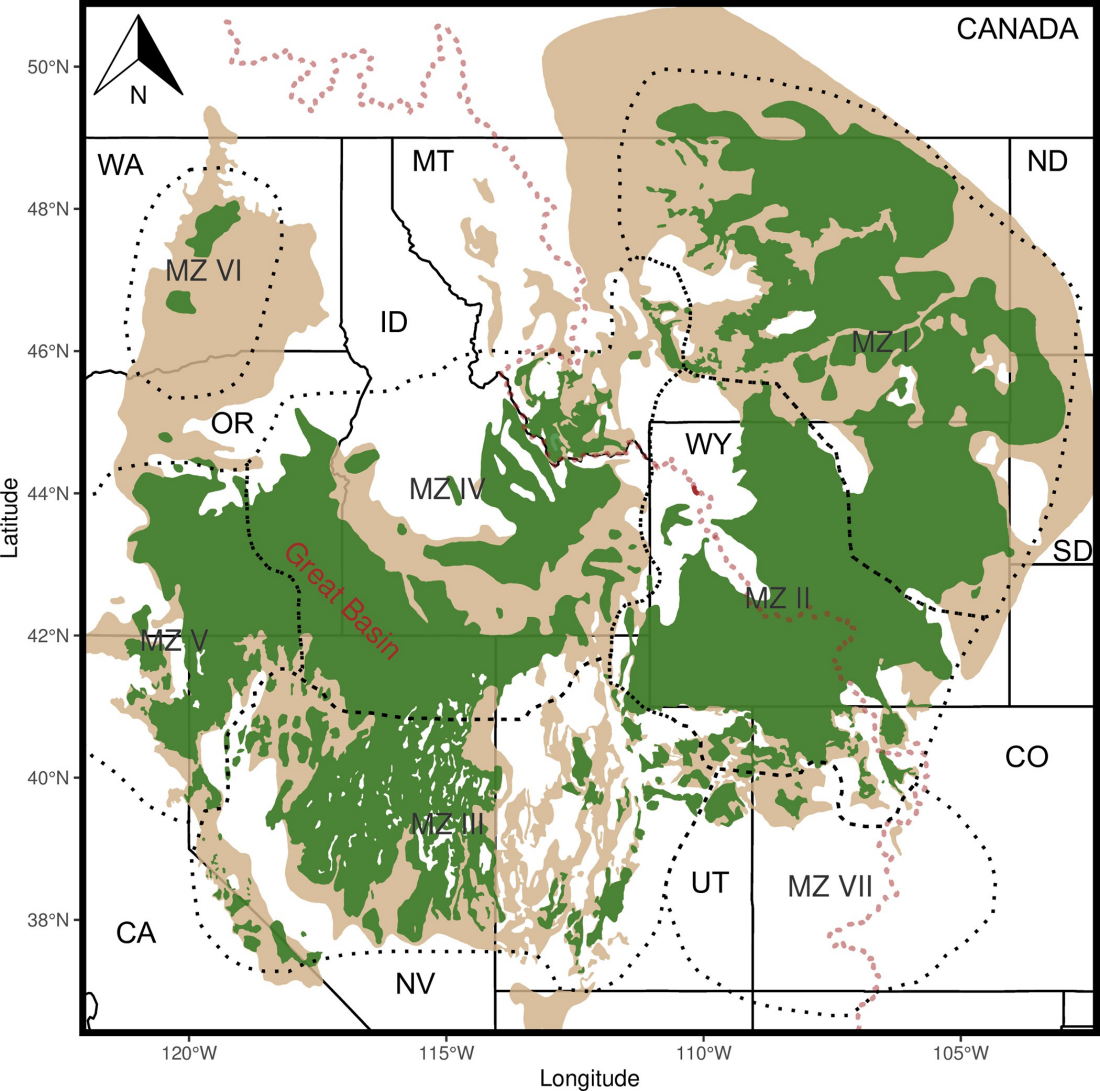
Current (dark green) and pre-settlement (tan) distribution of Greater Sage-grouse (*Centrocercus urophasianus*) in the western part of North America. (distribution layers available at https://www.sciencebase.gov/catalog/item/52e17ac3e4b0d0c3df9a3968 and https://www.sciencebase.gov/catalog/item/543d9947e4b0fd76af69cc74). The dotted red line represents the Continental Divide. Dotted black lines represent the seven sage-grouse management zones (MZ). State names are represented by the following abbreviations California (CA), Colorado (CO), Idaho (ID), Montana (MT), Nevada (NV), North Dakota (ND), Oregon (OR), South Dakota (SD), Utah (UT), Washington (WA), and Wyoming (WY).

Wide-ranging species residing in continuously distributed habitat pose substantial challenges for the characterization of genetic structure. Comprehensive sampling coverage for such a species requires a large number of individuals to be genotyped and the analysis of the genetic data to discern population structure is likely to be complicated by IBD. Here, we illustrate both the challenges inherent in and strategies for characterizing genetic structure in a wide-ranging species of significant conservation concern, the Greater Sage-grouse (*Centrocercus urophasianus*; sage-grouse hereafter). Although sage-grouse occupy sagebrush habitat covering much of western North America, their range has been reduced by 56% since pre-European settlement (Fig 1) leaving some populations small and isolated [21]. Sage-grouse management is moving towards a more coordinated approach that encompasses the entire species’ range [22–24] and could benefit from additional information on gene flow and population structure on similar scales. To overcome the difficulties of assessing sage-grouse genetic structure over such a large scale, we developed a novel method to identify centers of genetic differentiation and combine multiple methods identifying common genetic patterns that can be used to inform management and conservation strategies for this and other wide-ranging species. Using these approaches, we aimed to (1) describe large-scale patterns of population genetic structure and gene flow and (2) to characterize genetic subpopulation centers across the range of sage-grouse.

## Methods

### Genetic sampling

We collected 16,420 genetic samples across the range of sage-grouse in western North America spanning 11 U.S. states and two Canadian provinces. Feathers that had been dropped by sage-grouse during breeding activity were collected from 2005 to 2015. In addition, a small number of blood samples collected by other researchers as part of separate radio telemetry and GPS tracking field research were made available to us for use in this study. The spatial distribution of our sampling was optimized as described in Hanks et al. [25]. Briefly, following a pilot sample, we used a latent spatial model to identify spatial trends in genetic variation. We then identified geographic areas exhibiting the greatest transition in allele frequencies but where we lacked samples and increased our sample resolution in these areas in subsequent years.

### Microsatellite genotyping

The laboratory methods used to generate the genetic data for this study have been described previously [26, 27] and detailed methods are in S1 Appendix. Briefly, DNA was extracted and used in the amplification of 15 microsatellite loci, 12 of which were developed for sage-grouse [28–30]. We conducted extensive quality control to ensure the reliability of all genotypes and identified and removed multiple recaptures of the same individual. The data used in this study are available as a USGS data release [31].

### Sample optimization

We sampled some regions of the range with greater intensity than others. As heterogeneity in sampling intensity has been shown to affect the analysis of spatial genetic structure [18, 32, 33], we thinned the full dataset to achieve a more homogenous spatial representation. To thin the dataset, we followed the approach of Row et al. [27]. We used a hierarchical clustering approach to first define clusters, by grouping spatially proximate sampling locations using a hierarchical clustering analysis (*hclust* function with complete method) in R [34]. Each location was assigned to its own cluster, with the algorithm combining the two most proximal clusters iteratively until a single cluster remained. Distances between clusters were recomputed after each join by the Lance-Williams dissimilarity formula [35]. Finally, we used the *cutree* function in R to define clusters separated by a threshold distance of >50 km, representing two times the maximum average summer to winter movement distance for any population in Wyoming [36]. Once clusters were defined, we randomly chose ten individuals per cluster for those clusters that had >10 individuals.

We conducted a group-based principal component analysis (PCA) by computing the mean component scores for all individuals located within each state/province/regional group using the R packages *stats* and *gstudio* [37] and plotted the results using *ggplot2* [38]. Previous genetic and genomic research has shown that sage-grouse from the Bi-State region (along the border between California and Nevada in the southern part of the range in both states, Fig 1) and from Washington are substantially differentiated from other parts of the sage-grouse range and were likely isolated for long periods of time [39–42]. Therefore, we grouped all individuals from the Bi-State region together to separate them from other individuals from Nevada and California. Our group-based PCA revealed (see Results) that, as has been found in previous analyses, the Bi-State and the Washington sage-grouse, are significantly differentiated from all other sage-grouse (S1 Fig). Since we were interested in large-scale patterns of population structure and gene flow across the core of the range (i.e., not influenced by isolated peripheral populations that are known to be genetically distinct), we removed the Washington and Bi-State populations from subsequent analyses with one exception—the analysis of estimated effective migration rates (i.e., gene flow) across the entire range of sage-grouse.

### Examining population genetic structure

To investigate large-scale trends in genetic structure, we conducted an individual-based spatial principal component analysis (sPCA) calculated in the R package *adegenet* [43]. Additionally, we used the Bayesian clustering program STRUCTURE v2.3.4 [44] using the admixture model, correlated allele frequencies, and allele frequency distribution parameter (λ) set to 1. Analyses were run with non-informative priors and burn-in of 1,000,000 iterations followed by 1,000,000 Markov Chain Monte-Carlo (MCMC) iterations. For initial analysis, we completed ten replicates for each value of *K* from 1–30. The best-supported value of *K* was determined using two methods as implemented in STRUCTURE HARVESTER [45]. First, we plotted the mean and standard deviation of ln Pr(X|*K*) at each value of *K* and evaluated the point at which those values plateau. Second, we calculated and plotted ΔK following Evanno et al. [46]. Finally, we used CLUMPP v 1.1.2 [47] to average posterior probabilities of individual assignment to each of the *K* clusters across all ten STRUCTURE replicates. In CLUMPP we used 10,000 repeats of the greedy method with greedy option two and the pairwise matrix similarity statistic, *G*.

We examined spatial autocorrelation in our samples using the *mantel*.*correlog* function in the *vegan* package [48] inputting AMOVA genetic distance calculated using the *genetic_distance* function in R package *gstudio* [37]. We computed a multivariate Mantel correlogram (as described in [35]) with 80 distance classes, limiting the correlogram to the distance classes that included all points. A Pearson correlation was used in calculation of the Mantel statistic, with 999 permutations to test for significance, and a Bonferroni correction of P-values for multiple testing including progressive correction of multiple-testing (as described in [35]; briefly, the test for the first distance class: no correction, the test for the second distance class: correct for 2 simultaneous tests, the test for the k-th distance class: correct for k simultaneous tests).

### Identification of subpopulation centers

Sage-grouse are continually distributed over large portions of their range and the genetic structure of the species follows a pattern of IBD [49] across continuous habitats [42]. Consequently, in many parts of the range, discrete genetic boundaries likely do not exist and defining hard boundaries for genetic populations is problematic [18]. Therefore, our objective was to identify genetic subpopulation centers, which would theoretically represent the core of each distinct genetic group. To do this, we used two independent methods. Our first approach involved assigning individuals to a genetic group based on their maximum posterior probability score from the standard STRUCTURE analysis above, then using *adehabitatHR* functions *kernelUD* and *getverticeshr* in R to calculate the 25%, 50%, and 75% kernel density estimate (KDE) of these genetic groups based on the maximum population assignment. Our second approach used a spatial iterative bifurcation process (SIBP) composed of three steps for each iteration: (1) division of the individuals into two clusters, (2) spatial interpolation of individual posterior probabilities of membership for each of the two clusters, and (3) selection of individuals constituting the ’subpopulation center’ for each of the two clusters. In step one, we analyzed the entire sample at *K* = 2 using STRUCTURE and CLUMPP with all parameter settings as described above. In step two, we spatially interpolated the individual posterior probabilities (where values range from 0–1, and total 1) of individual subpopulation membership to each of the *K* = 2 clusters using the default settings of the *krig* function in the *fields* package [50] in R, following Fedy et al. [6]. The result of the fitted model is an interpolative surface derived from irregularly spaced data. From the kriged posterior probabilities of cluster membership we generated spatial isoclines of probability of membership to each of two subpopulations based on the spatial arrangement of samples and their respective strength of membership. In step three, we divided individuals into two groups based on the kriged isoclines such that all individuals within each of the two ≥ 0.70 isoclines were subset into their respective groups and retained. Samples located within the < 0.70 isoclines for either of the two clusters (i.e., not members of the two subpopulations or groups) were dropped from further iterations. We repeated this process of analysis, krig, subset for retained groups until no individuals remained within the ≥ 0.70 isoclines. In naming subpopulation centers, we switched between the binary nomenclature of 1|2 and a|b to indicate each subpopulation center’s lineage. For example, the primary subdivision of all samples was 1 and 2. The secondary subdivision was 1a|1b, and 2a|2b. We also assigned names to subpopulation centers based on geographic regions.

### Quantification of genetic diversity and divergence

We used *gstudio* [37] in R to characterize genetic diversity within each subpopulation center using the following metrics: observed (*H*_o_) and expected (*H*_e_) heterozygosity, average number of alleles (*A*), effective number of alleles (*Ae*), number of alleles with at least 5% frequency (*A*_*95*_), and the inbreeding statistic *F*_IS_. In GENEPOP v4.5.1 [51], we tested for deviations from Hardy-Weinberg proportions (HWP) and for gametic disequilibrium among loci correcting for multiple tests for significance using Bonferroni corrected P-values. We used GENODIVE [52] to quantify divergence among subpopulation centers using both *Ɵ*_ST_ [53] and Jost’s *D* [54].

### Estimated effective migration surfaces

We used the Estimating Effective Migration Surfaces program (EEMS; [55]) to quantify genetic differentiation resulting from differences in effective migration. To do this, we used EEMS to model rates of migration among demes using a stepping-stone approach [56]. The program overlays the sample range with a tight systematic grid and approximates the expected genetic differentiation among individuals under an IBD model. Then, comparing actual genetic differentiation among individuals to the IBD model, the program algorithms identify areas of divergence between actual and modeled differentiation. Those areas where actual differentiation is greater than predicted by the IBD model are areas of lower effective migration, whereas areas where actual differentiation is less than predicted by the IBD model are areas of greater effective migration. Using EEMS, we identified geographic areas across the species’ range with lower effective migration than expected under IBD (resistance to gene flow), with approximately equivalent effective migration (neutral effect on gene flow), or with greater effective migration (facilitation of gene flow). We plotted the EEMS-output spatial data layer using the *rEEMSplots* package [55] and the *ggplot2* package [38] in R. In EEMS, we fit models to, and averaged the results for, deme counts of 500 and 1,000, repeating this process three times at each deme count to ensure model convergence across random starting points. We used 1,000,000 MCMC iterations burn-in, 2,000,000 subsequent iterations, and thinned by 9,999 iterations. We repeated this analysis for the thinned dataset including Washington and the Bi-State populations.

## Results

### Microsatellite genotyping and sample thinning

Genotyping and quality control yielded 6,725 individuals from 1,364 breeding areas or leks (median = 3, IQR = 4, range = 1–62 individuals per lek) collected across the entire range from 2005 to 2015. The sample genotypes we used were the same as those refined for use in Cross et al. [26] and Row et al. [27], now including samples from Washington. After thinning of the data to avoid heterogeneous sample distribution, the number of individuals was reduced to 2,134 from 927 leks (median = 2, IQR = 2, range = 1–13 individuals per lek). Further removal of the individuals from the Bi-State population and Washington state resulted in 2,091 individuals from 915 leks (median = 2, IQR = 2, range = 1–13 individuals per lek).

### Determination of population genetic structure

**Fig 2:**
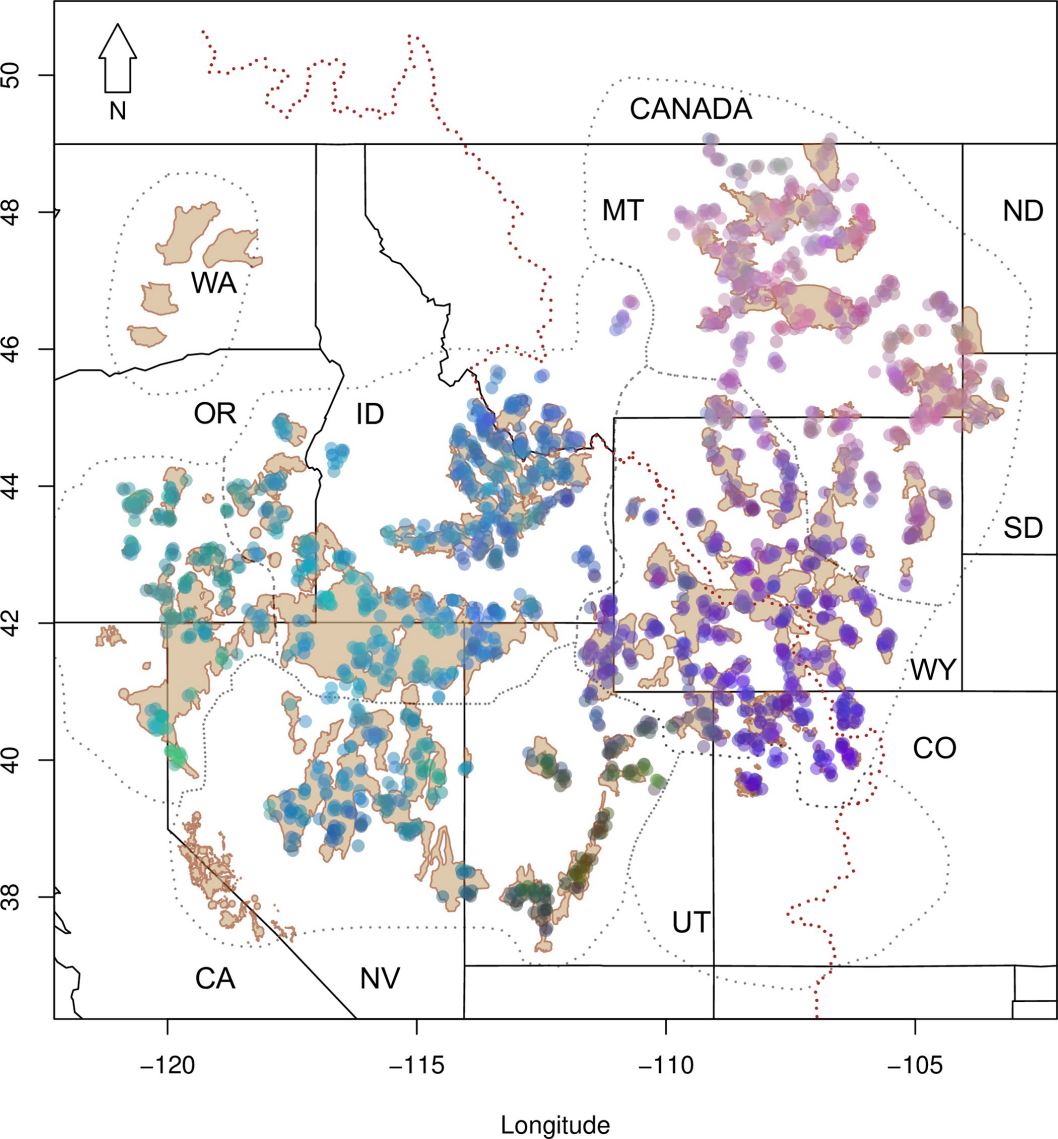
Spatial principal components analysis color plot of the first three eigenvectors for 15-locus microsatellite data from Greater Sage-grouse samples collected across the species’ range. The colors summarize the first three components by translating each score into a channel of color (red, green, and blue). The dotted red line represents the Continental Divide. Tan polygons represent Priority Areas for Conservation and dotted black lines represent the seven sage-grouse Management Zones (MZ). State names are represented by the following abbreviations California (CA), Colorado (CO), Idaho (ID), Montana (MT), Nevada (NV), North Dakota (ND), Oregon (OR), South Dakota (SD), Utah (UT), Washington (WA), and Wyoming (WY).

Our PCA grouped by state/province/region (S1 Fig) showed the populations in Washington and in the Bi-State were more divergent than average from other groups. Therefore, we withheld these individuals from all other analyses (except the second analysis of effective migration). In the sPCA, we retained eigenvalues 1–3, as they were the most divergent from the other axes and characterized the global trends in variance and spatial autocorrelation. The first eigenvalue split the northeast (Montana, North and South Dakota and northeastern Wyoming) and the remainder of the range (S2A Fig). The second eigenvalue highlighted a difference between southwestern Wyoming, Colorado, and Utah and the rest of the range (S2B Fig). The third eigenvalue separated Utah from much of the rest of the range, yet also showed regional differentiation in the Dakotas and southeastern Montana and the westernmost portion of the range (S2C Fig). When combined, these first three eigenvalues (Fig 2) did not reveal distinct population boundaries, but rather a pattern of population cores and transition zones between those cores. However, at least four groups are apparent: one group included most of Montana, North Dakota, South Dakota, and northeastern Wyoming (Fig 2; pink), which transitions to a differentiated group including southwestern Wyoming, northeastern Utah, and Colorado (Fig 2; purple). Further south, a shorter transition to the green and brown in central and southern Utah was detected (Fig 2). The remainder of the range—the greater Great Basin into Idaho and southwestern Montana—grouped together (Fig 2; turquoise).

In our standard STRUCTURE analysis, the most likely value of *K* given our data was equivocal. The several common approaches to selecting the most appropriate *K* each supported a different value. The point at which the mean ln Pr(X|*K*) began to plateau was approximately at *K* = 6 (S3A and S3C Fig). This was also the point at which the rate of change in ln Pr(X|*K*) began to diminish, and so it was the value of *K* for which we observed the second greatest Δ*K* statistic value (S3B Fig). However, it could also be argued that the values of ln Pr(X|*K*) plateaued at *K* = 12, and the maximum value occurred at *K* = 21 (S3A and S3D Fig). The Δ*K* indicated *K* = 2 had the greatest support as the uppermost hierarchical level of structure (S3B Fig). At *K* = 6, individuals were divided into groups that were mostly contiguous, such that most geographically proximal individuals were assigned to the same genetic cluster (S4A Fig), with the six groups representing (1) the greater Great Basin (Oregon, California, Nevada), (2) Idaho and southwestern Montana, (3) central and southern Utah, (4) southwestern/central Wyoming, Colorado, and northeastern Utah (5) northeastern Wyoming with the Dakotas and southeastern Montana, and (6) central/northern Montana. As in the sPCA, contact zones between groups showed increased admixture within individuals compared to those closer to group centroids. Individual assignment was particularly ambiguous for individuals throughout much of Nevada and the Great Basin. At *K* = 12, geographic discreteness was reduced with multiple groups co-occurring (S4B Fig).

We observed statistically significant positive autocorrelation among pairs of sage-grouse samples separated by < 50 km. Some, but not all, distance classes above 450 km showed significant negative autocorrelation. Pairwise samples between 50 and 450 km were not significantly autocorrelated.

### Identification of subpopulation centers

**Fig 3:**
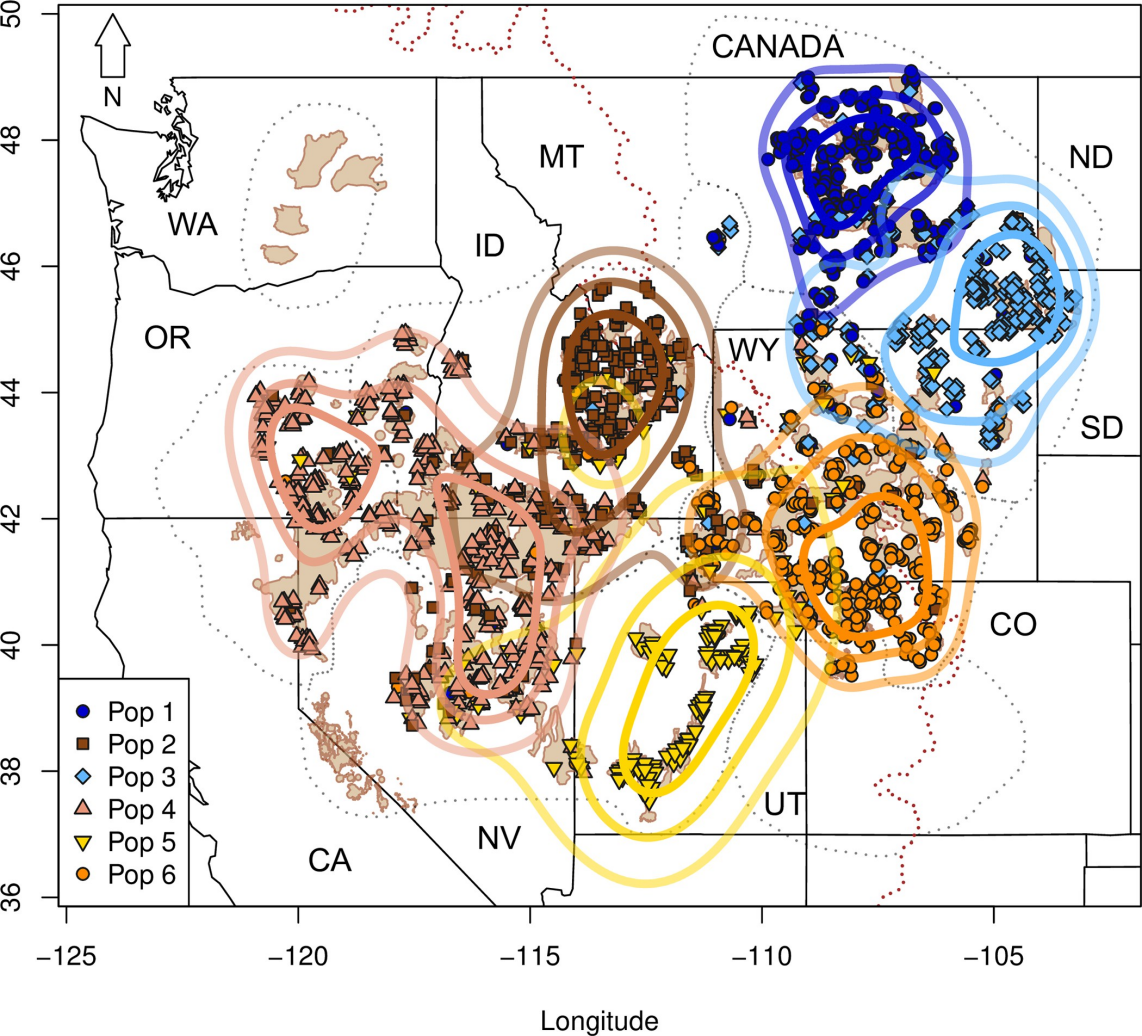
Boundaries of Greater Sage-grouse subpopulation centers at *K* = 6 from a STRUCTURE analysis using a 25% (darkest polygon), 50% (medium polygon), and 75% (lightest polygon) kernel density estimate to determine genetically distinct groups. The six different colors represent the six clusters identified in the STRUCTURE analysis. The dotted red line represents the Continental Divide. Tan polygons represent Priority Areas for Conservation and dotted black lines represent the seven sage-grouse management zones (MZ). State names are represented by the following abbreviations California (CA), Colorado (CO), Idaho (ID), Montana (MT), Nevada (NV), North Dakota (ND), Oregon (OR), South Dakota (SD), Utah (UT), Washington (WA), and Wyoming (WY).

We identified 25%, 50%, and 75% KDE for individuals assigned to each of the *K* = 6 genetic clusters (Fig 3). The KDE revealed non-overlapping but non-contiguous (25% KDE) or largely non-overlapping and contiguous (50% KDE) areas that included one group in northeastern Montana (dark blue; Fig 3), a second including southeastern Montana, northeastern Wyoming, North and South Dakota (light blue; Fig 3), a third including southwestern Wyoming, Colorado, and a small portion of Utah (orange; Fig 3), a fourth including most of Utah and a sliver of southwestern Wyoming (yellow; Fig 3), a fifth covering much of Nevada, Oregon, southwestern Idaho, and extreme northeastern California (salmon; Fig 3), and a sixth that included central Idaho and southwestern Montana, dipping slightly into northeastern Nevada and northwestern Utah (brown; Fig 3). The 75% KDE revealed contact zones between groups. Three groups converged in southwestern Wyoming along the border where Wyoming, Utah and Idaho converge, (brown, orange, yellow) and south-central Idaho (salmon, brown, and yellow).

**Fig 4:**
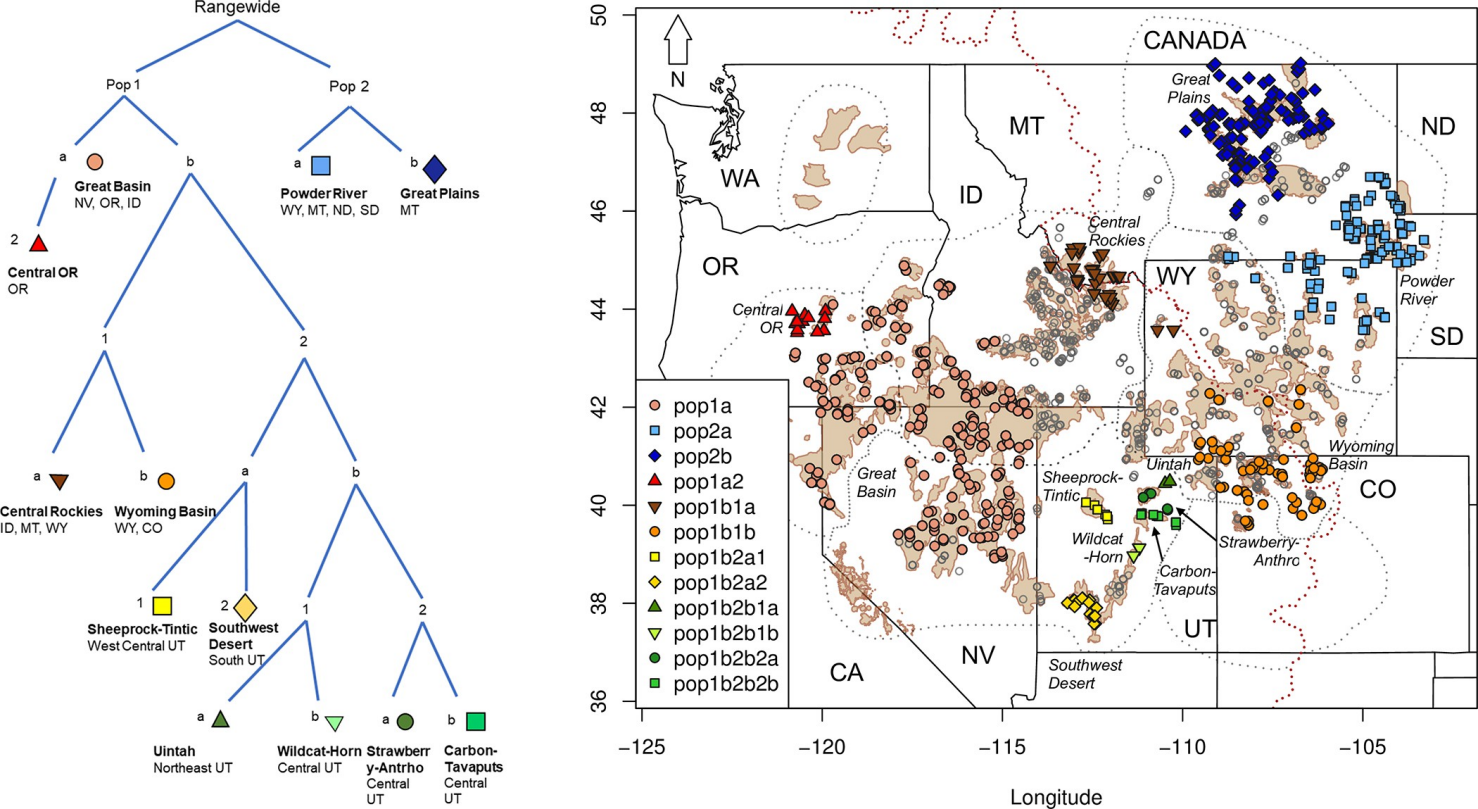
(A) Diagram showing how Greater Sage-grouse subpopulation centers were identified using 6 rounds of spatial iterative bifurcation process (for detailed interpolated maps and a summary of individuals included in each bifurcation, see S5 and S6 Figs) and (B) the distribution of individuals in each subpopulation center with color and symbol representing the 12 subpopulation centers. Open grey symbols represent individuals that occur outside of the subpopulation centers. The dotted red line represents the Continental Divide. Tan polygons represent Priority Areas for Conservation and dotted black lines represent the seven sage-grouse management zones (MZ). State names are represented by the following abbreviations California (CA), Colorado (CO), Idaho (ID), Montana (MT), Nevada (NV), North Dakota (ND), Oregon (OR), South Dakota (SD), Utah (UT), Washington (WA), and Wyoming (WY).

We discovered twelve nested subpopulation centers across the species’ contiguous range after six successive rounds of analysis (Fig 4) using our SIBP method. The primary division in genetic structure loosely aligned near the Continental Divide (S5A and S6A Figs) bifurcating the range into eastern and western groups (Pop 1 and Pop 2, Fig 4A). Secondary subdivisions (S5B and S6B Figs) divided the western group (Pop1, Fig 4A) into a Great Basin group (Great Basin Pop1a, Fig 4A), and a group east of the Great Basin (Pop 1b, Fig 4A), and separated the eastern group (Pop 2, Fig 4A) into a north central Montana group (Great Plains Pop 2b, Fig 4A) and a group covering the Dakotas, southeastern Montana, and eastern Wyoming (Powder River Pop 2a, Fig 4A). Tertiary subdivision (S5C and S6C Figs) bifurcated a group in Oregon (Central OR Pop1a2, Fig 4A) from the rest of western Great Basin (from which no kriged posterior probabilities were ≥0.70) and divided the group east of the Great Basin into two groups (Pop 1b1 and Pop 1b2, Fig 4A). Quaternary subdivision (S5D and S6D Figs) separated a group in southwestern Montana, eastern Idaho, western Wyoming, northern Utah (Central Rockies Pop1b1a, Fig 4A), from one in northwestern Colorado and southern Wyoming (Wyoming Basin Pop1b1b, Fig 4A) as well as two groups in central Utah (Pop 1b2a and 1b2b, Fig 4A). Quintenary (S5E and S6E Figs) and senary (S5F and S6F Figs) subdivisions further bifurcated the central Utah group into six subpopulation centers (Fig 4A). Each successive round of substructure analysis resulted in a cut of 3–52% of the total starting samples with a mean cut of 19% (those individuals not within the ≥0.70 isocline S1 Table). After seven rounds of analysis (the senary round), no individuals remained within the 0.70 isocline (S1 Table).

### Quantification of genetic diversity

Among all samples, there was an average of 16.53 alleles per locus ranging from eight at TUT3 to 33 at MSP11, with an expected heterozygosity of 0.845, and an *F*_IS_ of 0.075 (Table 1). After grouping samples by SIBP, no single locus was out of HWP in more than one subpopulation center; however, there were four loci in the Great Basin group, one in the Central Rockies group, two in the Powder River group, and one in the Great Plains group that were out of HWP. No locus pairs were in significant gametic disequilibrium in more than one subpopulation center. Within subpopulation centers the average number of alleles per locus ranged from 4.87 in Wildcat-Horn to 13.60 in the Great Basin subpopulation center (mean = 8.77 ± 3.44 [SD]). Across all subpopulation centers, *H*_*e*_ averaged 0.747 ± 0.16 [SD] (Table 1).

**Table 1:** Measures of genetic diversity across 15 microsatellite loci within each of the spatial iterative bifurcation process (SIBP) subpopulation centers detected for Greater sage-grouse.

Subpopulation center name (number)	*n*	*A*	*Ae*	*A*_*95*_	*H*_*o*_	*H*_*e*_	*F*_IS_
All Samples	2091	16.53	7.40	6.60	0.782	0.845	0.075
Great Basin (1a)	458	13.60	7.32	6.80	0.802	0.843	0.049
Central OR (1a2)	39	8.53	5.24	5.80	0.787	0.790	0.001
Central Rockies (1b1a)	70	11.47	6.59	6.93	0.805	0.832	0.030
Wyoming Basin (1b1b)	125	11.80	6.16	5.87	0.787	0.818	0.038
Sheeprock-Tintic (1b2a1)	18	5.80	3.54	4.67	0.731	0.689	-0.067
Southwest Desert (1b2a2)	25	6.47	3.70	4.47	0.657	0.712	0.077
Uintah (1b2b1a)	10	5.00	3.43	5.00	0.720	0.668	-0.077
Wildcat-Horn (1b2b1b)	10	4.87	3.38	4.87	0.724	0.669	-0.083
Strawberry-Anthro (1b2b2a)	15	6.40	4.06	4.80	0.733	0.721	-0.016
Carbon-Tavaputs (1b2b2b)	15	5.80	3.71	4.47	0.705	0.703	0.005
Powder River (2a)	220	12.67	5.59	5.80	0.766	0.793	0.033
Great Plains (2b)	248	12.87	5.57	5.47	0.778	0.801	0.029

Within the table, *n* = sample size, *A* = average number of alleles across 15 loci, *Ae* = effective number of alleles (the # of equally frequent alleles required to achieve the same *H*_e_), *A*_*95*_ = number of alleles with at least 5% frequency, *H*_o_ = observed heterozygosity, *H*_e_ = expected heterozygosity, *F*_IS_ = 1-(*H*_o_/*H*_e_)–a measure of departure from Hardy-Weinberg proportions (HWP) within groups/subpopulations (positive values indicate a deficit of heterozygotes, negative values indicate an excess of heterozygotes).

Divergence among the 12 subpopulation centers ranged widely (*Ɵ*_ST_: 0.021–0.156, Jost’s D: 0.084–0.444; S2 Table). Divergence (S2 Table) measured using *Ɵ*_ST_ was greatest among subpopulation centers in Utah (Uintah, Wildcat-Horn, Sheeprock-Tintic, and Southwest Desert). Yet when using Jost’s D, divergence was greatest between the Great Plains subpopulation center and centers in Utah (Sheeprock-Tintic, Uintah, Wildcat-Horn), between the Central Rockies and Wildcat-Horn and between centers in Utah (Sheeprock-Tintic and Uintah). Divergence was least when measured using *Ɵ*_ST_ between the subpopulation centers in Oregon (Central OR and Great Basin) and when measured using Jost’s D between the centers in Montana (Great Plains and Powder River).

### Estimated effective migration surfaces

**Fig 5:**
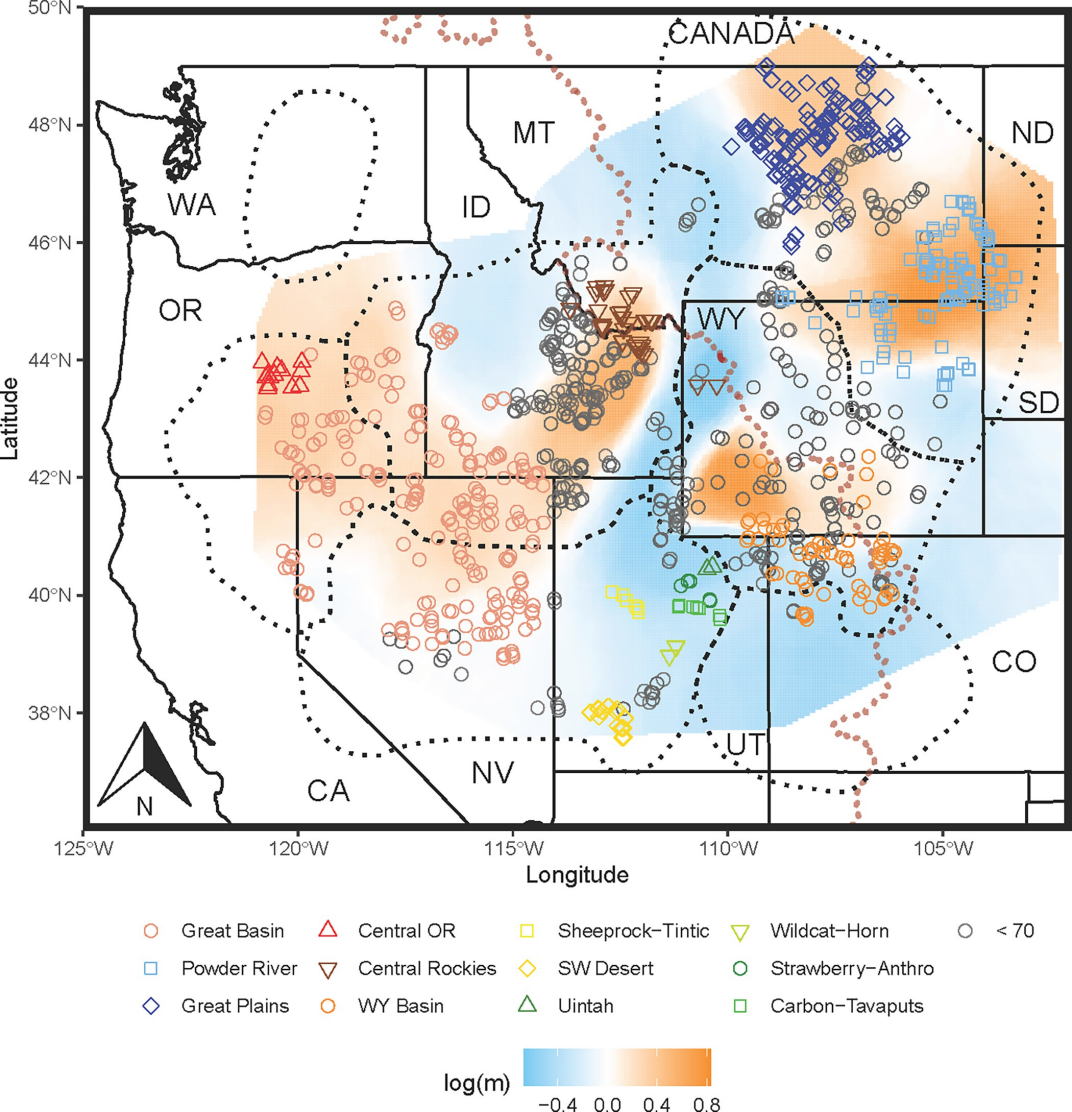
The 12 Greater Sage-grouse subpopulation centers identified using the spatial iterative bifurcation process overlaid on the effective migration surface. The color and symbol represent each of the 12 subpopulation centers. Open grey symbols represent individuals outside of subpopulation centers. Effective migration rates are shown on a log scale where the zero value (white) indicates the mean effective migration rate, positive values (orange) indicate greater than average effective migration and negative values (blue) indicate those less than average. The dotted red line represents the Continental Divide. Tan polygons represent Priority Areas for Conservation and dotted black lines represent the seven sage-grouse management zones (MZ). State names are represented by the following abbreviations California (CA), Colorado (CO), Idaho (ID), Montana (MT), Nevada (NV), North Dakota (ND), Oregon (OR), South Dakota (SD), Utah (UT), Washington (WA), and Wyoming (WY).

We estimated effective migration rates for the entire range of sage-grouse including Washington and the Bi-State, and then separately excluding samples from those two areas. As expected, analysis of the entire range revealed areas of constrained migration between Washington and the rest of the range and to a lesser extent between the Bi-State population and the rest of the range (S7 Fig). When excluding Washington and the Bi-State, effective migration was greatest within the Powder River (southeastern Montana and the Dakotas), diminishing slightly moving northward into the Great Plains (Fig 5, positive log migration, orange). Other areas of greater migration include within the northern part of the Wyoming Basin subpopulation center, the Central OR subpopulation center, and stretching across the Great Basin connecting the Great Basin subpopulation center to the Central Rockies subpopulation center. Low migration rates (Fig 5, negative log migration, shaded blue) were found in northern Idaho, between eastern Montana (Great Plains and Powder River subpopulation centers) and southwestern Montana (Central Rockies subpopulation center), through western Wyoming, extending south through far eastern Idaho, into all but the northwesternmost corner of Utah, and all of Colorado. Additional areas of low migration included central Wyoming between the northern part of the Powder River subpopulation center and the southern portion of the Wyoming Basin center stretching south into Colorado.

## Discussion

Wildlife management and conservation strategies benefit from an understanding of genetic structure and connectivity among populations. Many species of conservation concern have small ranges occurring in discrete patches of habitat that can be described genetically through relatively straightforward analyses. However, the genetic structure of wide-ranging species (whether of conservation concern or of management interest) is more difficult to assess due to analytical difficulties both from the sheer quantity of data and the underlying issue of IBD. To overcome these challenges, we developed a novel method (SIBP) designed specifically for continuously distributed species. We illustrate its utility by identifying centers of genetic variation and comparing multiple additional analytical methods to reveal common patterns of genetic structure and connectivity in sage-grouse. Further, we show how the information gleaned from these analyses can be used to guide management and conservation actions.

### Comparing patterns among analyses

Sage-grouse habitat is contiguous in some areas of the range and fragmented and isolated in others (Fig 1) and the extent to which IBD might impact our analysis was unknown, although a previous genetic study suggested that IBD is a prominent pattern across the range [42]. Although we used STRUCTURE and sPCA to define major patterns of genetic difference and to identify centers of genetic differentiation through SIBP (all of which are influenced by IBD), we also used EEMS that was designed specifically for instances in which broad-scale IBD exists and compared all results.

All analyses supported differentiation and low effective migration between an east and west group that split loosely along the Continental Divide (Figs 2 and 5), except where the topographical abruptness of the Divide is less severe (e.g., between southwestern Montana and eastern Idaho, and within WY). Divisions below the initial east/west split varied by analysis with sPCA resolving the fewest groups followed by STRUCTURE and SIBP. Within the eastern group, northern Montana (Great Plains) and southeastern Montana/Dakotas/northeastern Wyoming (Powder River) were further separated by STRUCTURE (Fig 3, S4A Fig) and the SIBP (Fig 4), yet only minimally with sPCA (shown as differentiated with the 3^rd^ eigenvector). Our EEMS analysis indicated slightly elevated effective migration between these two groups (Fig 5), which is consistent with the spatially predicted connectivity in that area described in Row et al. [27]. Within the western group, three or four additional groups were defined using sPCA and STRUCTURE with additional divisions detected using SIBP. Idaho and a Nevada/Oregon group comprise two (STRUCTURE) or three genetic (SIBP) groups (Figs 3 and 4), yet sPCA revealed only subtle differentiation and EEMS showed elevated effective migration among these groups (Figs 2 and 5). The Wyoming Basin group (southwestern Wyoming and Colorado) was consistently recognized as one group throughout all analyses. Effective migration within this group varied substantially, however, with high migration in southwestern Wyoming and lower migration in Colorado (Fig 5). In contrast, there was no consistency in the classification of samples in Jackson Hole, Wyoming (isolated population in the northwestern part of the state). This group clusters with samples from Idaho and Montana (Central Rockies subpopulation center) in the SIBP (Fig 4), it aligns with southwestern Wyoming in the sPCA (Fig 2), and it lies outside all polygons in the KDE (Fig 3). This population occurs in an area of local low effective migration in the EEMS analysis (Fig 5) and has been found to be isolated previously [57] yet not as genetically distinct as Washington or the Bi-State [39]. The degree of differentiation and isolation in central and southern Utah was also unclear but was nested in classification (i.e., the group was either considered one large group or six small groups within it) unlike Jackson Hole (which was assigned to completely different groups depending on the analysis). The SIBP analysis (Figs 4 and 5) revealed 6 subpopulation centers in Utah with increased differentiation (S2 Table), whereas the standard STRUCTURE analysis (Fig 3, S4A Fig) and to a lesser extent spatial PCA (Fig 2) showed one group differentiated from the rest of the range. Sage-grouse in central and southern Utah occur in small, isolated patches of sagebrush likely highly affected by genetic drift which has led to increased differentiation. Translocations from multiple areas in Utah (including the northeast and northwest) into central Utah (specifically into Strawberry-Anthro) complicate the interpretation of the structure found here [58–60] and may explain why the differentiation in the Strawberry-Anthro subpopulation center was reduced and its genetic diversity greater than others in that area.

### Improving resolution and extent

Our analysis provides a comprehensive view of population genetic structure and gene flow across the entire range of sage-grouse. Past evaluations of connectivity for sage-grouse have examined population structure and gene flow both range-wide [42] and at smaller scales [5, 6, 40, 57, 61–64]. The only other range-wide assessment of population structure included over 1,000 samples largely from hunter-harvested birds, yet the spatial resolution was limited as samples were consolidated by hunt unit or county [42]. We found general agreement with that study yet found greater differentiation in central Utah (6 subpopulation centers here vs. 3 clusters prior), and greater connectivity in Colorado (1 subpopulation center here vs. 2 clusters prior). These differences are most likely due to increased resolution and spatial continuity of sampling in this study but could be due to shifts in genetic structure over time, as ~20 years (6–10 generations) have passed since the original study.

While our results are similar to those of others conducted at the state level or larger scales, there are subtle differences which are likely due to differing study extents. This study incorporated the genotypes used in Fedy et al. [6] who examined population structure in Wyoming and Cross et al. [63] who did similar research in Montana and the Dakotas. In most of our analyses we documented two or three groups as opposed to the four found previously in Wyoming [6]. Importantly, this study documents a prominent division between northeastern and southwestern Wyoming representing a previously unrecognized east-west split separating all sage-grouse into two main groups (Fig 5, S2A, S4A and S7 Figs). This division was largely along the boundary between management zone (MZ)1 and MZ2 (Figs 1 and 3). In Montana and the Dakotas, we found the same three groups as Cross et al. [63], yet they found additional substructure in their hierarchical analysis some of which can be discerned in our STRUCTURE analysis at K = 12 (S4B Fig). This analysis of sage-grouse genetic variation range-wide, at high resolution, and with sampling homogeneity removes artifacts that may be introduced by irregular sampling or truncation of sampling extent at state and provincial boundaries rather than biological boundaries such as a species’ distribution.

### Incorporation with other range-wide connectivity products

This research builds upon two recent studies that used the same data set to examine different aspects of connectivity. The first developed a genetic network for sage-grouse, characterized the network connectivity structure, and identified nodes serving as hubs of genetic connectivity such that these nodes could be prioritized based on their importance for maintaining network structure and connectivity [26]. The second evaluated functional connectivity by determining the specific factors driving genetic divergence within sage-grouse MZs, determining the landscape and habitat features that most impact gene flow within individual zones [27]. When individual resistance models were stitched together across MZs, a wide-ranging functional connectivity surface was produced [27]. This study takes the next step in evaluating connectivity by describing and quantifying large-scale patterns of population genetic structure and gene flow.

In the network study Cross et al. [26] found that breeding areas in the C. J. Strike Reservoir Watershed in southwestern Idaho, and in the Bighorn Lake and Upper Green-Slate watersheds in southwestern and north central Wyoming respectively, were important for maintaining genetic connectivity range-wide. These regions exhibited elevated effective migration rates (Fig 5), yet only one (C. J. Strike Reservoir) was within a center of subpopulation differentiation, indicating that areas outside of subpopulation centers are likely important for maintaining overall connectivity, while the subpopulation centers serve as generators of and reservoirs for genetic variation. Our map of effective migration was in general agreement with the functional connectivity map from Row et al. [27], yet the spatial patterns inferable from our map are much coarser as our effective migration map is based solely on effective migration rates (the pattern) and not based on the landscape variables (the process) driving that pattern. Nonetheless, our areas of greatest effective migration concur with those of greatest functional connectivity described in Row et al. [27]. This is of particular note in central Wyoming east of the Continental Divide where we found lower than expected migration rates and Row et al. [27] found constrained functional connectivity between MZs 1 and 2 that defined the east-west division of sage-grouse. In this same region, Cross et al. [26] also identified lek clusters essential to maintaining range-wide connectivity (hubs of connectivity identified in the top 1% of betweenness centrality). Our findings of lowered effective migration in Utah and Colorado also mirrored the work of Row et al. [27] who detected diminished functional connectivity as evidenced by fewer and/or more diffuse modeled gene flow.

### Relating genetic structure to current management boundaries

The distribution and connectivity of sage-grouse have been subdivided previously based on biologically relevant criteria. For example, floristic zones were used to define the seven MZs [65] and the populations and subpopulations delineated by Connelly et al. [66] used a combination of habitat, lek locations, and the degree of presumed fragmentation. More recent work has defined hierarchical clusters based on seasonal habitat and sage-grouse movements [22–24]. While our genetic groups are consistent with some of these boundaries (e.g., MZ1 and MZ2) our findings suggest less population subdivision than suggested by the boundaries for MZs 3, 4, or 5 (our Great Basin genetic subpopulation center [Fig 5] spans all three of these MZs). Differences between the subdivisions described here and in other efforts may lie in the fact that genetic data measures functional movement: dispersal followed by reproduction, rather than structural connectivity based on habitat distribution and Euclidean distance. Several studies have examined whether sage-grouse males or females disperse farther with conflicting results, possibly due to study extent and the number of individuals marked [62, 67–69]. If males disperse farther than females and fail to reproduce, their movements are irrelevant to gene flow. As such, our connectivity assessment and characterization of subpopulation centers may differ from those based purely on habitat characteristics and movement data yet are important for management. As genetic variation and connectivity are vital for the ability to cope with current and future environmental stochasticity and anthropogenic alterations, understanding how genes flow across the landscape is a vital component of sage-grouse conservation.

### Implications for sage-grouse management

Previous studies have highlighted the genetic distinctiveness in the Bi-State and the Washington populations suggesting that these groups may warrant being managed separately [39, 42], and we reaffirmed that here. We identified more subtle subdivision across the rest of the range of sage-grouse that was formerly unknown including a distinct east-west split generally along the Continental Divide. To a large extent most of our analyses recognized two groups in the east (Powder River and Great Plains) and at least four in the west (Wyoming Basin, greater Great Basin, Central Rockies, and Utah), which could be used to help define large scale management units. The 6 higher-order subpopulation centers of differentiation that resulted from the first three rounds of SIBP are core areas of differentiation that may be important to protect to maintain maximum genetic diversity across the range. The 6 finest scale subpopulation centers, occurring in the fragmented habitat in Utah, are areas with smaller numbers of isolated sage-grouse that could be monitored to ensure that genetic diversity in those areas is maintained or increased and to ensure that connectivity with other subpopulation centers is not lost. Additionally, the areas outside of subpopulation centers where different genetic groups converge (e.g., south-central Idaho and southwestern Wyoming) could be priorities for maintaining overall connectivity, especially where they overlap areas previously identified as important to sage-grouse connectivity [26, 27]. The data and analyses presented provide a baseline for monitoring future changes in sage-grouse connectivity and genetic diversity resulting from landscape changes.

## Conclusion

Increased anthropogenic disturbance and changing climates threaten to further fragment the habitat of wide-ranging, continuously distributed species. Understanding genetic connectivity and taking management action to minimize or mitigate those threats to it is essential for the persistence of such species. In concert, our novel SIBP approach and the process of leveraging multiple different analyses to find common genetic patterns can provide the comprehensive, large scale genetic information needed to make effective management decisions.

## Supporting information

S1 Appendix (DOCX)

S1 Fig Group-based principal components analysis of Greater Sage-grouse samples from across the species’ range genotyped at 15 microsatellite loci. Mean principal component scores were calculated by state/province of origin with the Bi-State (samples along the border between California (CA) and Nevada (NV)) considered as a separate group from the rest of the samples collected in CA and NV, and separate from those collected in Canada (CAN_SASK), Oregon (OR), Utah (UT), Idaho (ID), Washington (WA), Colorado (CO), Wyoming (WY), Montana (MT), North Dakota (ND), South Dakota (SD).
(TIF)

S2 Fig First (A), second (B), and third (C) principal components (eigenvalues) of the spatial principal components analysis of Greater sage-grouse across their range derived from analysis of 15 microsatellite loci. White squares represent negative spatial principal component values, and black squares represent positive spatial principal component values. Square size represents the absolute magnitude of the value. The dotted red line represents the Continental Divide. State names are represented by the following abbreviations California (CA), Colorado (CO), Idaho (ID), Montana (MT), Nevada (NV), North Dakota (ND), Oregon (OR), South Dakota (SD), Utah (UT), Washington (WA), and Wyoming (WY).
(TIF)

S3 Fig Mean and standard deviation of ln Pr(X|*K*) at each value of *K* (A) Δ*K* values for each successive increase in *K* (B) STRUCTURE plots at *K* = 6 (C) and *K* = 12 (D) for the standard STRUCTURE analysis of Greater Sage-grouse genotyped across their range using 15 microsatellites. We used the admixture model, correlated allele frequencies, and set the allele frequency distribution parameter to 1 with a burnin and number of Markov Chain Monte Carlo repetitioins set to 1,000,000. Ten replicates were run for each value of *K* from 1–30.
(TIF)

S4 Fig The predominant genetic assignment for each individual Greater Sage-grouse from STRUCTURE analysis at A) *K* = 6 and 12) *K* = 12 based on the maximum posterior probability. The dotted red line represents the Continental Divide. State names are represented by the following abbreviations California (CA), Colorado (CO), Idaho (ID), Montana (MT), Nevada (NV), North Dakota (ND), Oregon (OR), South Dakota (SD), Utah (UT), Washington (WA), and Wyoming (WY).
(TIF)

S5 Fig Interpolated maps generated from the spatial iterative bifurcation process (SIBP) for Greater Sage-grouse. The SIBP involved kriging the posterior probability of population membership to each of *K* = 2 clusters following CLUMPP to average across multiple independent STRUCTURE runs. Open circles show individuals within the 70% isocline for population membership to one of the two populations. Figures depict populations: 1 and 2 (A); 1a, 1b, 2a, 2b (B), 1a1, 1a2, 1b1, 1b2 (C), 1b1a, 1b1b, 1b2a, 1b2b (D), 1b2a1, 1b2a2, 1b2b1, 1b2b2 (E), 1b2b1a, 1b2b1b, 1b2b2a, 1b2b2b (F). In naming subpopulations, we switched between the binary nomenclature of 1|2 and a|b to trace each subpopulation’s origin. For example, the primary subdivision of all sample was subpopulation 1 and 2. The secondary subdivision was 1a, 1b, 2a, and 2b.
(TIF)

S6 Fig Summary of the individual Greater Sage-grouse remaining within the 70% isocline after each successive round of the spatial iterative bifurcation process. After the first round, two population centers remained ([A] upper left defined by population 1 and 2). After the second round, each population was divided into another two subpopulations ([B] top right defined by pop1a, pop1b, pop2a, and pop2b). Four more rounds of analysis are represented in order by the [C-F].
(JPG)

S7 Fig Effective migration rate estimated the entire Greater sage-grouse data set including Washington and the Bi-state (samples collected along the border between California and Nevada). Effective migration rates are shown on a log scale where the zero value indicates the mean effective migration rate, positive values indicate greater than average effective migration (orange) and negative values indicate those less than average (blue). These maps represent the average of models run with a deme count of 500 and 1,000. State names are represented by the following abbreviations California (CA), Colorado (CO), Idaho (ID), Montana (MT), Nevada (NV), North Dakota (ND), Oregon (OR), South Dakota (SD), Utah (UT), Washington (WA), and Wyoming (WY).
(TIF)

S1 Table Number of Greater Sage-grouse individuals retained from each round of the spatial iterative bifurcation process (SIBP) analysis following STRUCTURE and CLUMPP analysis for *K* = 2, kriging, and retaining of those samples contained within the ≥ 0.70 subpopulation cluster membership isoclines (i.e., subpopulation centers). The primary round of analysis included 2,091 individuals. Subpopulation center nomenclature is shown across the top row of the table and the round of SIBP analysis is in the first column. After seven rounds of analysis, no samples remained within ≥ 0.70 population cluster membership kriged isoclines.
(XLSX)

S2 Table *Ɵ*_ST_ values (above the diagonal) and Jost’s D values (below the diagonal) among all pairs of Greater Sage-grouse subpopulation centers as defined by the hierarchical Bayesian clustering and kriging analysis. The centers are represented by both a number and a subpopulation center name as defined in Fig 4.
(XLSX)
