# Supplementary material for: New strategies for characterizing genetic structure in wide-ranging, continuously distributed species: A Greater Sage-grouse case study
Source: PLoS One. 2022 Sep 13;17(9):e0274189. doi: 10.1371/journal.pone.0274189 (PMC9469985; doi:10.1371/journal.pone.0274189)
Supplement: S1 Appendix — (DOCX) [file pone.0274189.s001.docx]

**S1 Appendix**

**DNA extraction**

We conducted genetic analysis at two different molecular biology laboratories: the Molecular Ecology Lab at the U.S. Geological Survey Fort Collins Science Center (FORT-MEL) and the National Genomics Center for Wildlife and Fish Conservation at the U.S. Forest Service Rocky Mountain Research Station (NGC). We established distinct protocols at the outset of the study to ensure consistency in laboratory genotyping and we detail them below. We extracted DNA from the quill (calamus) of each feather using the QIAGEN DNeasy Blood and Tissue Kit and the user-developed protocol for purification of total DNA from nails, hair, or feathers. The protocol was modified incubating samples for a minimum of 8 h to maximize tissue lysis, and by eluting DNA with 100 μl of Buffer AE to increase the final DNA concentration in the eluate. We extracted blood samples following the QIAGEN DNeasy Blood and Tissue Kit protocol for nucleated blood. At FORT-MEL, portions of the DNA extraction process were automated using a QIAcube (QIAGEN).

**Microsatellite DNA amplification and genotyping**

We used a panel of 15 highly polymorphic microsatellite loci (BG6, SGMS06.4, SGMS06.6, SGMS06.8, MSP11, MSP18, SG28, SG29, SG36, SG39, SGCA5, SGCA11, SGCTAT1, TUT3, and TUT4) to characterize genetic diversity, structure, and divergence. Primer information, PCR conditions, and electrophoresis at FORT-MEL and NGC are detailed in Cross et al. [1, 2] and Row et al. [3]. Amplification of microsatellite loci were conducted in eight multiplex polymerase chain reactions (PCR). At FORT-MEL, PCR products were visualized by first multi-loading based on primer label and PCR product size and combined with GeneScan LIZ 600 internal size standard (Applied Biosystems) and then electrophoresed through a capillary gel matrix on a AB3500 Automated DNA sequencer (Applied Biosystems). Allele sizes were based on the fragment length of each microsatellite locus using the software GeneMapper v4.1 (Applied Biosystems). Details for PCR product visualization and genotype calling at NGC are described in detail in Cross et al. [1].

Microsatellite genotyping in different laboratories can be problematic and requires calibration of allele sizes to ensure consistency in the data [4]. To deal with this issue, we conducted extensive quality control to ensure the reliability of all genotypes used in the analyses and consistency across the two laboratories. Over the course of many years, FORT-MEL developed and optimized a panel of fifteen microsatellites for use with feather samples [5-7] using genetic samples collected previously across the range of sage-grouse [8]. NGC was initially using a different set of microsatellite primers (only four of which were in common with those used by FORT-MEL). When the research efforts were joined, NGC continued using the four loci that the two laboratories had in common and added the 11 loci that FORT-MEL had developed and was already using. For calibration, FORT-MEL sent NGC 10 already genotyped samples and their fragment lengths (genotype calls). These 10 samples were selected to include the diversity of alleles known at each locus (all common and some rare alleles representing 50-100% of known alleles). NGC used the FORT-MEL genotyping calls to standardize their allele calls to be consistent with FORT-MEL. NGC had to shift allele calls after genotyping for only the four loci already in use. To later test for allele calling consistency, 151 samples were independently amplified and genotyped across the entire 15-locus microsatellite panel by each laboratory. Fifty-seven of these 151 samples genotyped successfully enough to compare allele calls; these represented unique individuals. All retained genotypes aligned between the two laboratories in accordance with the prior calibration. Throughout the genotyping process, FORT-MEL and NGC thoroughly compared chromatographs, gel images, and allele frequency distributions for each locus to ensure that genotyping calls were consistent. At both laboratories, all samples were PCR-amplified twice across the 15 microsatellite loci to screen for allele dropout, stutter artifacts, and false alleles [9] and at least two independent observers scored each sample. If any locus failed to amplify in either replicate or if there was a discrepancy between locus genotypes as scored by the two observers, PCR amplification and genotyping were repeated twice more. If a genotype was confirmed by this repeat analysis, then it was retained. If a genotype failed again, the sample was assigned a missing data score at the failed locus.

We removed any individual for which amplification failed at one-third or more of the loci (i.e., ≥ five loci). Subsequently, we screened genotypes to ensure consistency between allele length and microsatellite repeat motif length using MICROCHECKER v2.2.3 [10](Van Oosterhout et al. 2004). To identify and remove multiple captures of the same individual and to screen for genotyping error, we used DROPOUT 2.3 [11](as implemented in Schwartz et al. [12]), MICROCHECKER v2.2.3 [10], and package ALLELEMATCH 2.5 [13] in program R 3.3.0 [14] setting the guessOptimum parameter to TRUE. To evaluate all possible duplicates, we re-examined the raw data and removed those that represented duplicates once genotyping error had been corrected. The only exception to this was samples that were recaptured on a different lek, which were retained. Finally, we quantified the power of our microsatellite locus panel to discern individuals using probability identity (PI; [15]) which calculated the probability that two individuals drawn at random from the population have the same genotype across all loci in GENALEX [16]. The PI for 10 loci (we required an individual to have at least 10 loci genotyped to be included) was 1.66 X 10^-^^16^, which demonstrates our ability to uniquely identify individuals.

We retained all spatio-temporally recaptured individuals for all analyses, as we considered these recaptures reflective of genetic structure and connectivity. Upon completion of our analyses, we discovered two mistakenly retained spatio-temporal duplicates in the thinned data set that did not meet our retention criterion (0.09% of samples) and an additional four in the full data set (0.09% of samples). To evaluate the impact of these duplicates, we reran both the spatial PCA and the PCA by state and found no major differences in reported results. Given their minimal effect, we chose to leave these duplicates in the completed analysis and results rather than reconducting all analyses.

Any use of trade, firm, or product names is for descriptive purposes only and does not imply endorsement by the U.S. Government.

**References**

1. Cross TB, Naugle DE, Carlson JC, Schwartz MK. Hierarchical population structure in greater sage-grouse provides insight into management boundary delineation. Conserv Genet. 2016;17: 1417-33. <https://link.springer.com/article/10.1007/s10592-016-0872-z>

2. Cross TB, Naugle DE, Carlson JC, Schwartz MK. Genetic recapture identifies long-distance breeding dispersal in Greater Sage-Grouse (*Centrocercus urophasianus*). Condor: Ornith Appl. 2017;119: 155-66. <https://doi.org/10.1650/CONDOR-16-178.1>

3. Row JR, Oyler-McCance SJ, Fike JA, O'Donnell MS, Doherty KE, Aldridge CL, et al. Landscape characteristics influencing the genetic structure of greater sage-grouse within the stronghold of their range: A holistic modeling approach. Ecol Evol. 2015;5: 1955-69. <https://doi.org/10.1002/ece3.1479> PMID: 26045948

4. Sort M, Manuzzi A, Jiménez-Mena B, Ovenden J, Holmes B, Bernard A, et al. Come together: calibration of tiger shark (Galeocerdo cuvier) microsatellite databases for investigating global population structure and assignment of historical specimens. Conserv Genet Res. 2021;13: 209-20. <https://link.springer.com/article/10.1007/s12686-021-01197-5>

5. Taylor S, Oyler‐McCance S, Quinn T. Isolation and characterization of microsatellite loci in Greater Sage‐Grouse (Centrocercus urophasianus). Mol Ecol Notes. 2003;3: 262-4. <https://doi.org/10.1046/j.1471-8286.2003.00424.x>

6. Oyler-McCance SJ, St John J. Characterization of small microsatellite loci for use in non invasive sampling studies of Gunnison Sage-Grouse (Centrocercus minimus). Conserv Genet Res. 2010;2: 17-20. <https://link.springer.com/article/10.1007/s12686-009-9122-8>

7. Fike JA, Oyler-McCance SJ, Zimmerman SJ, Castoe TA. Development of 13 microsatellites for Gunnison Sage-grouse (Centrocercus minimus) using next-generation shotgun sequencing and their utility in Greater Sage-grouse (Centrocercus urophasianus). Conserv Genet Res. 2015;7: 211-4. <https://link.springer.com/article/10.1007/s12686-014-0336-z>

8. Oyler-McCance SJ, Taylor S, Quinn T. A multilocus population genetic survey of the greater sage‐grouse across their range. Mol Ecol. 2005;14: 1293-310. <https://doi.org/10.1111/j.1365-294X.2005.02491.x> PMID: 15813771

9. DeWoody J, Nason JD, Hipkins VD. Mitigating scoring errors in microsatellite data from wild populations. Mol Ecol Notes. 2006;6: 951-7. <https://doi.org/10.1111/j.1471-8286.2006.01449.x>

10. Van Oosterhout C, Hutchinson WF, Wills DP, Shipley P. MICRO‐CHECKER: software for identifying and correcting genotyping errors in microsatellite data. Mol Ecol Notes. 2004;4: 535-8. <https://doi.org/10.1111/j.1471-8286.2004.00684.x>

11. McKelvey K, Schwartz M. Dropout: a program to identify problem loci and samples for noninvasive genetic samples in a capture‐mark‐recapture framework. Mol Ecol Notes. 2005;5: 716-8. <https://doi.org/10.1111/j.1471-8286.2005.01038.x>

12. Schwartz MK, Cushman SA, McKelvey KS, Hayden J, Engkjer C. Detecting genotyping errors and describing American black bear movement in northern Idaho. Ursus. 2006;17: 138-48.

13. Galpern P, Manseau M, Hettinga P, Smith K, Wilson P. Allelematch: an R package for identifying unique multilocus genotypes where genotyping error and missing data may be present. Mol Ecol Res. 2012;12: 771-8. <https://doi.org/10.1111/j.1755-0998.2012.03137.x>

14. Team RC. R: A language and environment for statistical computing [Computer software manual]. Vienna, Austria2016.

15. Evett IW, Weir BS. Interpreting DNA evidence: statistical genetics for forensic scientists: Sinauer Associates Sunderland, MA; 1998.

16. Peakall R, Smouse PE. GENALEX 6: genetic analysis in Excel. Population genetic software for teaching and research. Mol Ecol Notes. 2006;6: 288-95. <https://doi.org/10.1111/j.1471-8286.2005.01155.x>
