# Supplementary figures and images for: New strategies for characterizing genetic structure in wide-ranging, continuously distributed species: A Greater Sage-grouse case study
Source: PLoS One. 2022 Sep 13;17(9):e0274189. doi: 10.1371/journal.pone.0274189 (PMC9469985; doi:10.1371/journal.pone.0274189)

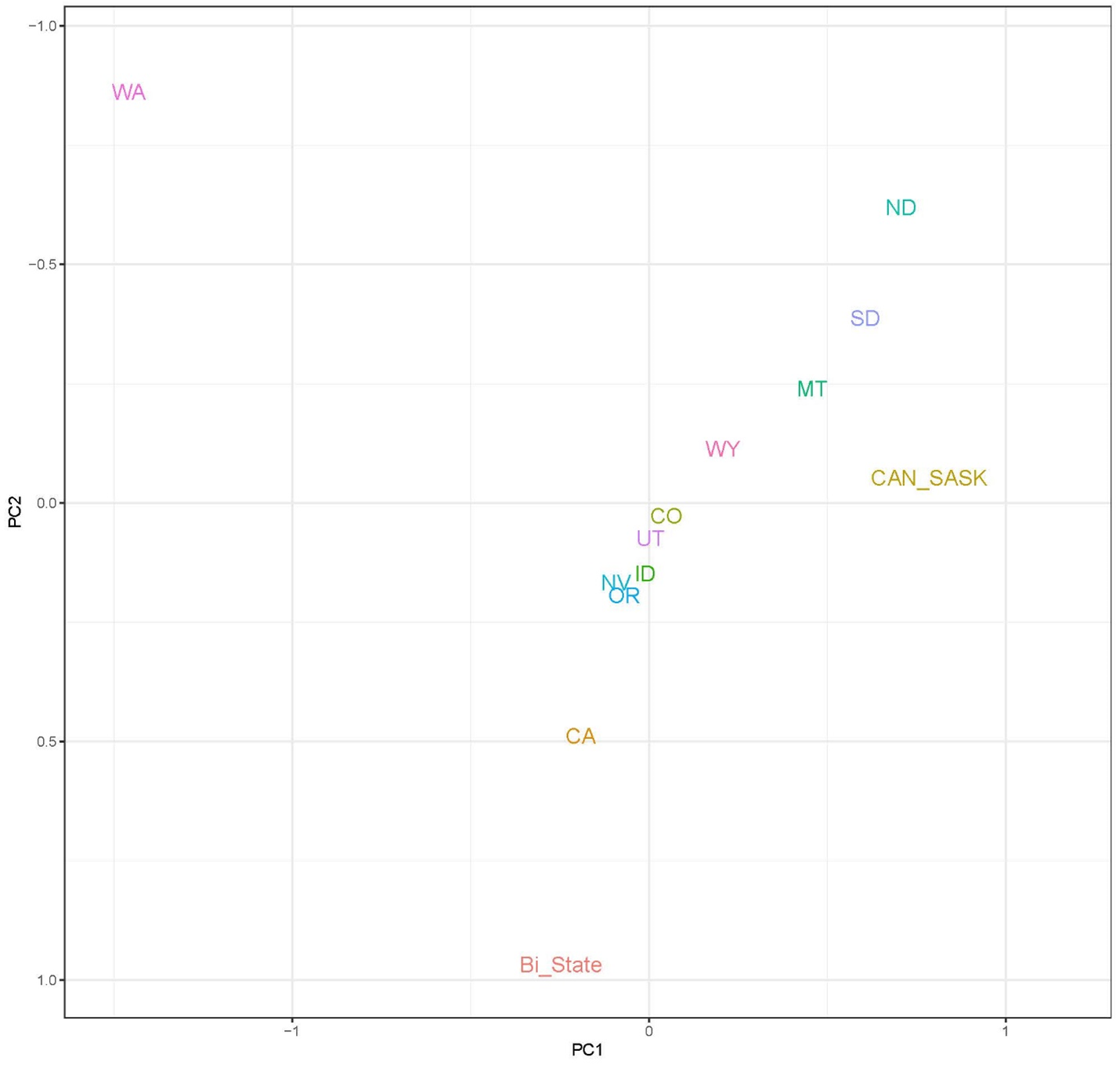

Supplement: S1 Fig — Mean principal component scores were calculated by state/province of origin with the Bi-State (samples along the border between California (CA) and Nevada (NV)) considered as a separate group from the rest of the samples collected in CA and NV, and separate from those collected in Canada (CAN_SASK), Oregon (OR), Utah (UT), Idaho (ID), Washington (WA), Colorado (CO), Wyoming (WY), Montana (MT), North Dakota (ND), South Dakota (SD). (TIF) [file pone.0274189.s002.tif]

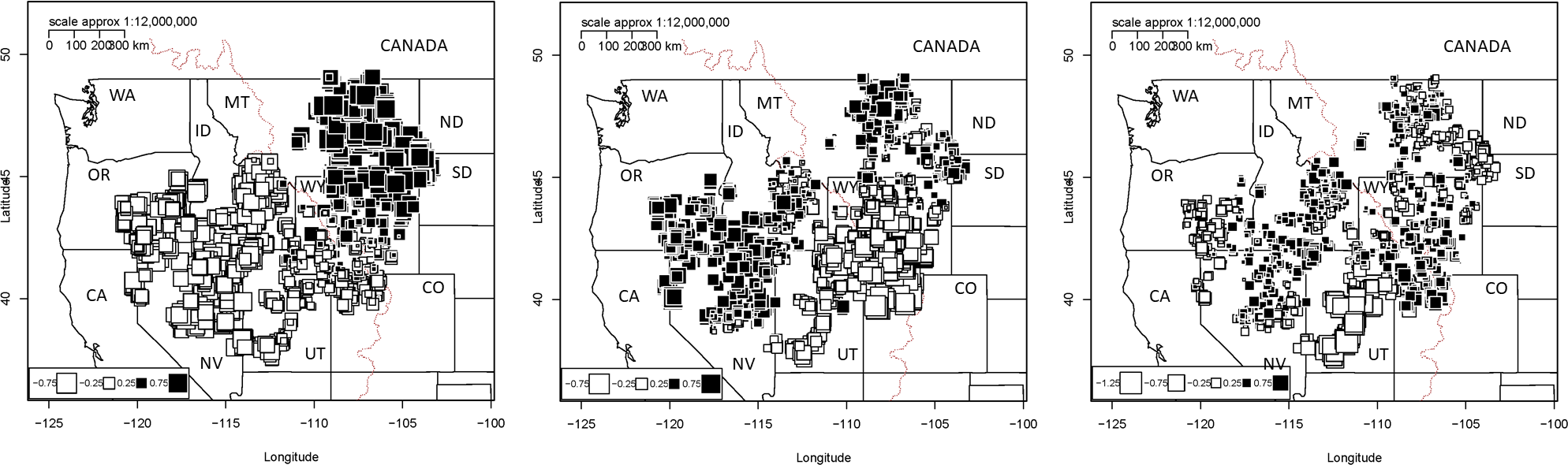

Supplement: S2 Fig — First (A), second (B), and third (C) principal components (eigenvalues) of the spatial principal components analysis of Greater sage-grouse across their range derived from analysis of 15 microsatellite loci. White squares represent negative spatial principal component values, and black squares represent positive spatial principal component values. Square size represents the absolute magnitude of the value. The dotted red line represents the Continental Divide. State names are represented by the following abbreviations California (CA), Colorado (CO), Idaho (ID), Montana (MT), Nevada (NV), North Dakota (ND), Oregon (OR), South Dakota (SD), Utah (UT), Washington (WA), and Wyoming (WY). (TIF) [file pone.0274189.s003.tif]

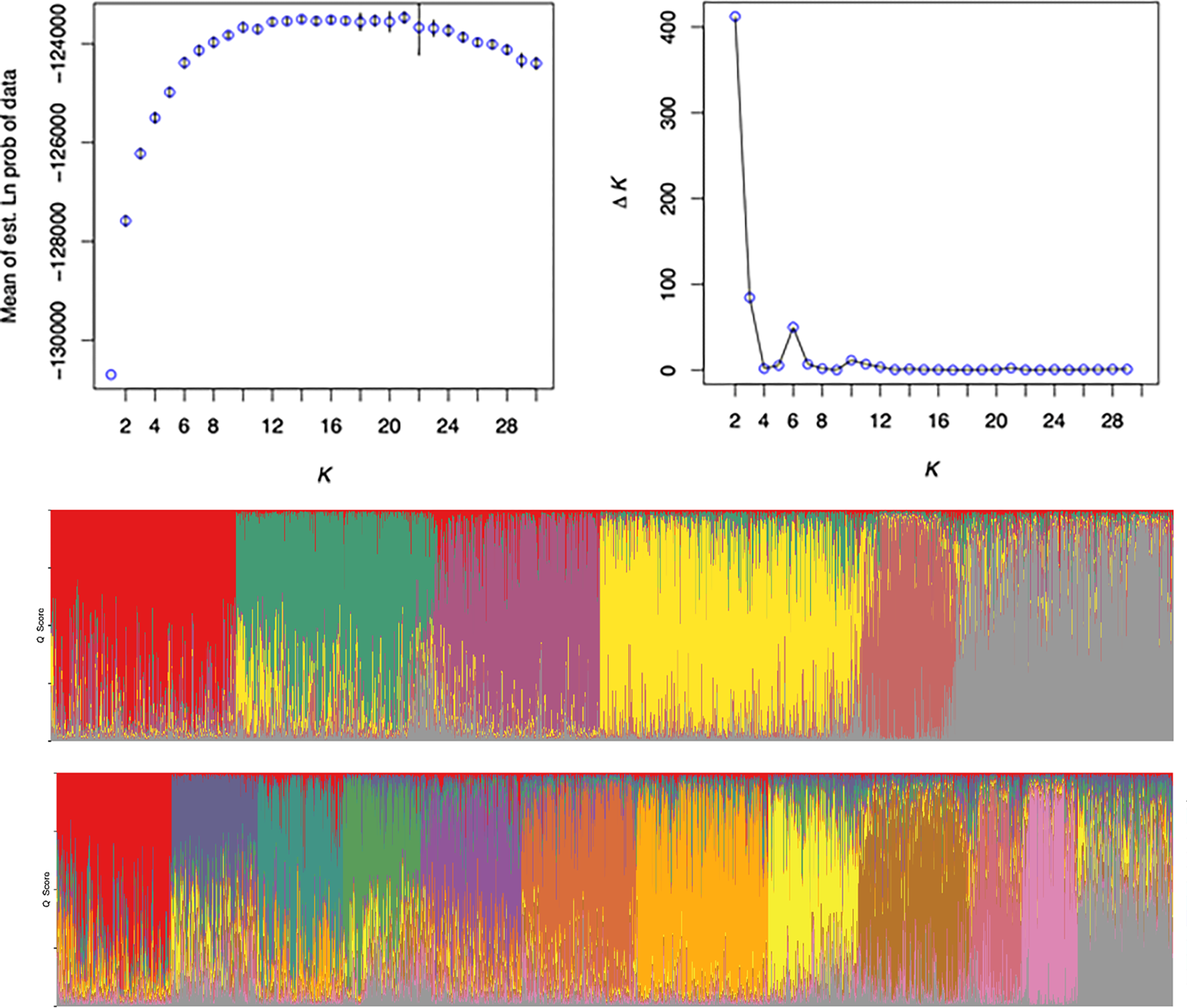

Supplement: S3 Fig — Mean and standard deviation of ln Pr(X|K) at each value of K (A) ΔK values for each successive increase in K (B) STRUCTURE plots at K = 6 (C) and K = 12 (D) for the standard STRUCTURE analysis of Greater Sage-grouse genotyped across their range using 15 microsatellites. We used the admixture model, correlated allele frequencies, and set the allele frequency distribution parameter to 1 with a burnin and number of Markov Chain Monte Carlo repetitioins set to 1,000,000. Ten replicates were run for each value of K from 1–30. (TIF) [file pone.0274189.s004.tif]

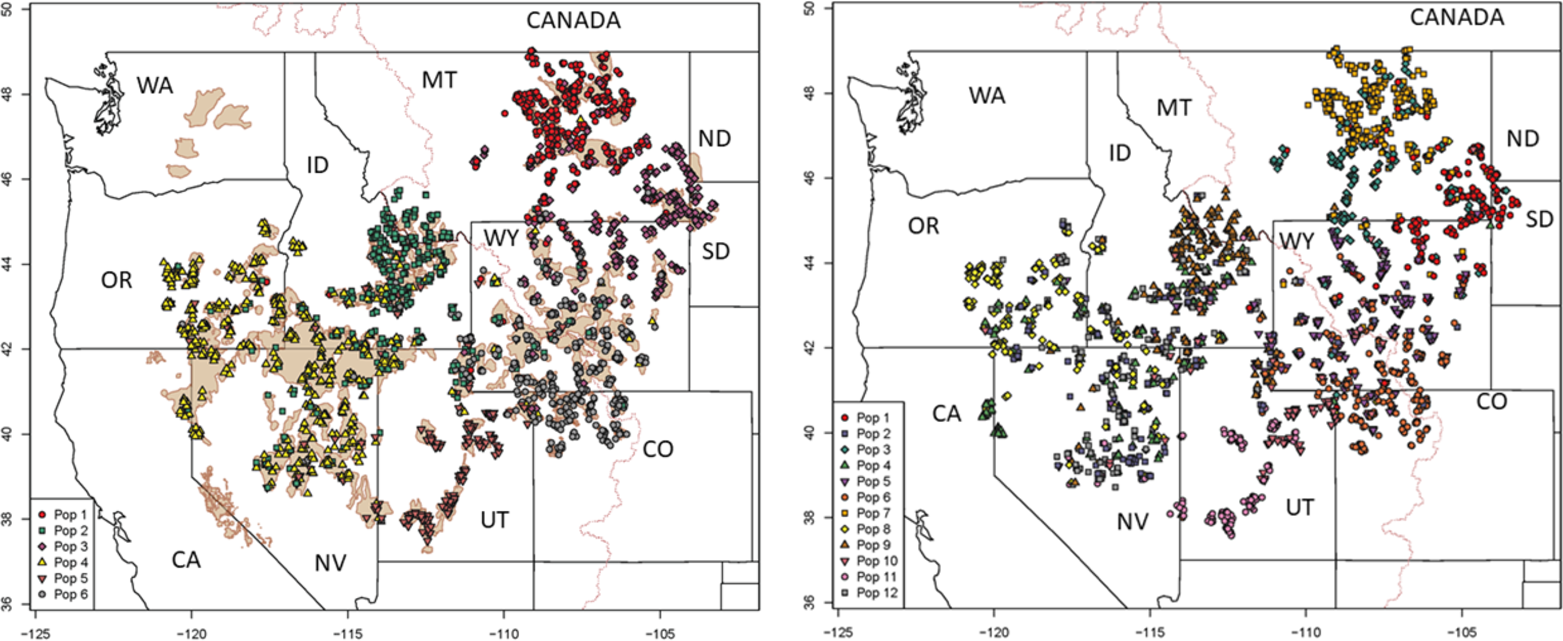

Supplement: S4 Fig — The dotted red line represents the Continental Divide. State names are represented by the following abbreviations California (CA), Colorado (CO), Idaho (ID), Montana (MT), Nevada (NV), North Dakota (ND), Oregon (OR), South Dakota (SD), Utah (UT), Washington (WA), and Wyoming (WY). (TIF) [file pone.0274189.s005.tif]

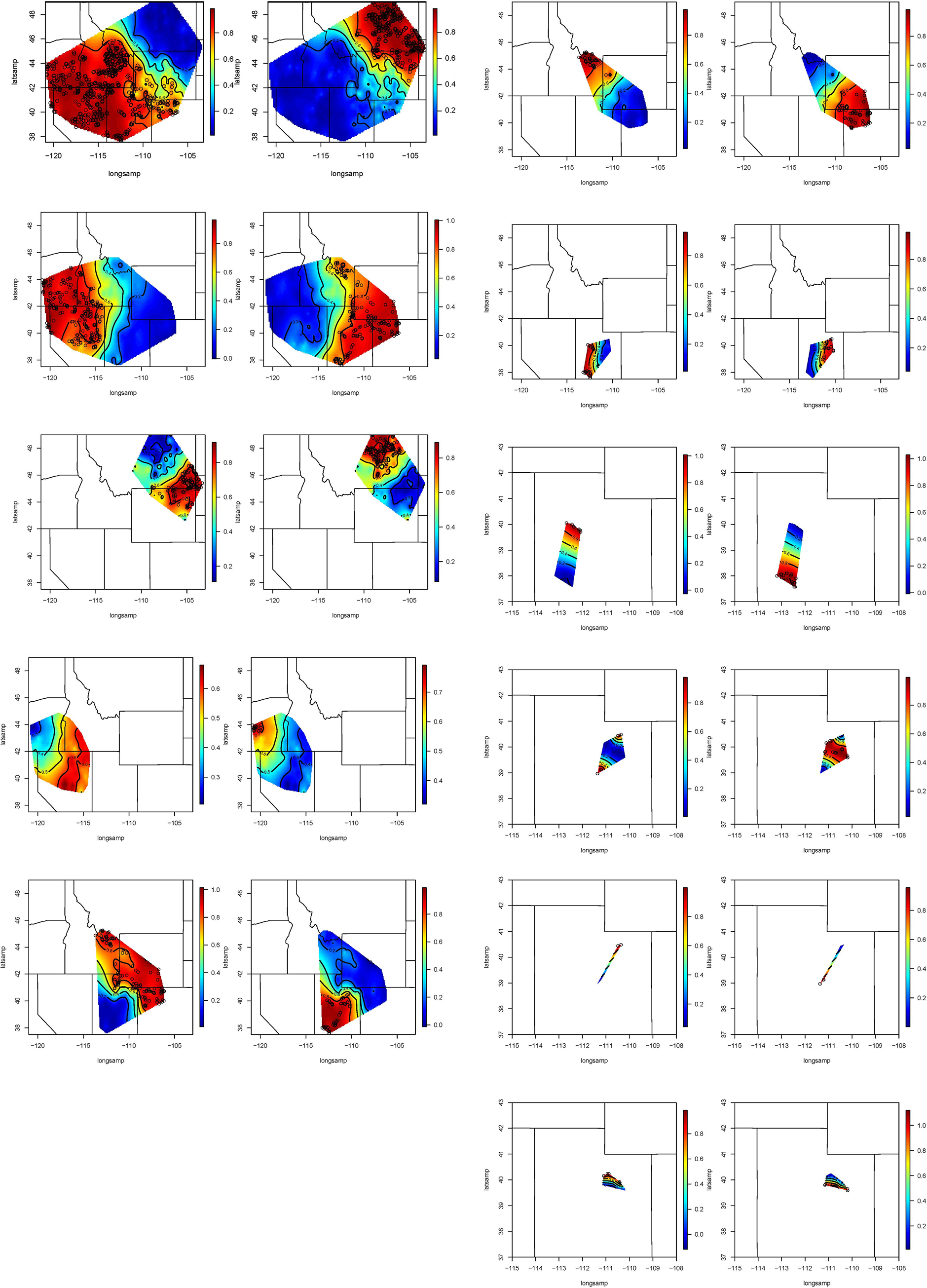

Supplement: S5 Fig — The SIBP involved kriging the posterior probability of population membership to each of K = 2 clusters following CLUMPP to average across multiple independent STRUCTURE runs. Open circles show individuals within the 70% isocline for population membership to one of the two populations. Figures depict populations: 1 and 2 (A); 1a, 1b, 2a, 2b (B), 1a1, 1a2, 1b1, 1b2 (C), 1b1a, 1b1b, 1b2a, 1b2b (D), 1b2a1, 1b2a2, 1b2b1, 1b2b2 (E), 1b2b1a, 1b2b1b, 1b2b2a, 1b2b2b (F). In naming subpopulations, we switched between the binary nomenclature of 1|2 and a|b to trace each subpopulation’s origin. For example, the primary subdivision of all sample was subpopulation 1 and 2. The secondary subdivision was 1a, 1b, 2a, and 2b. (TIF) [file pone.0274189.s006.tif]

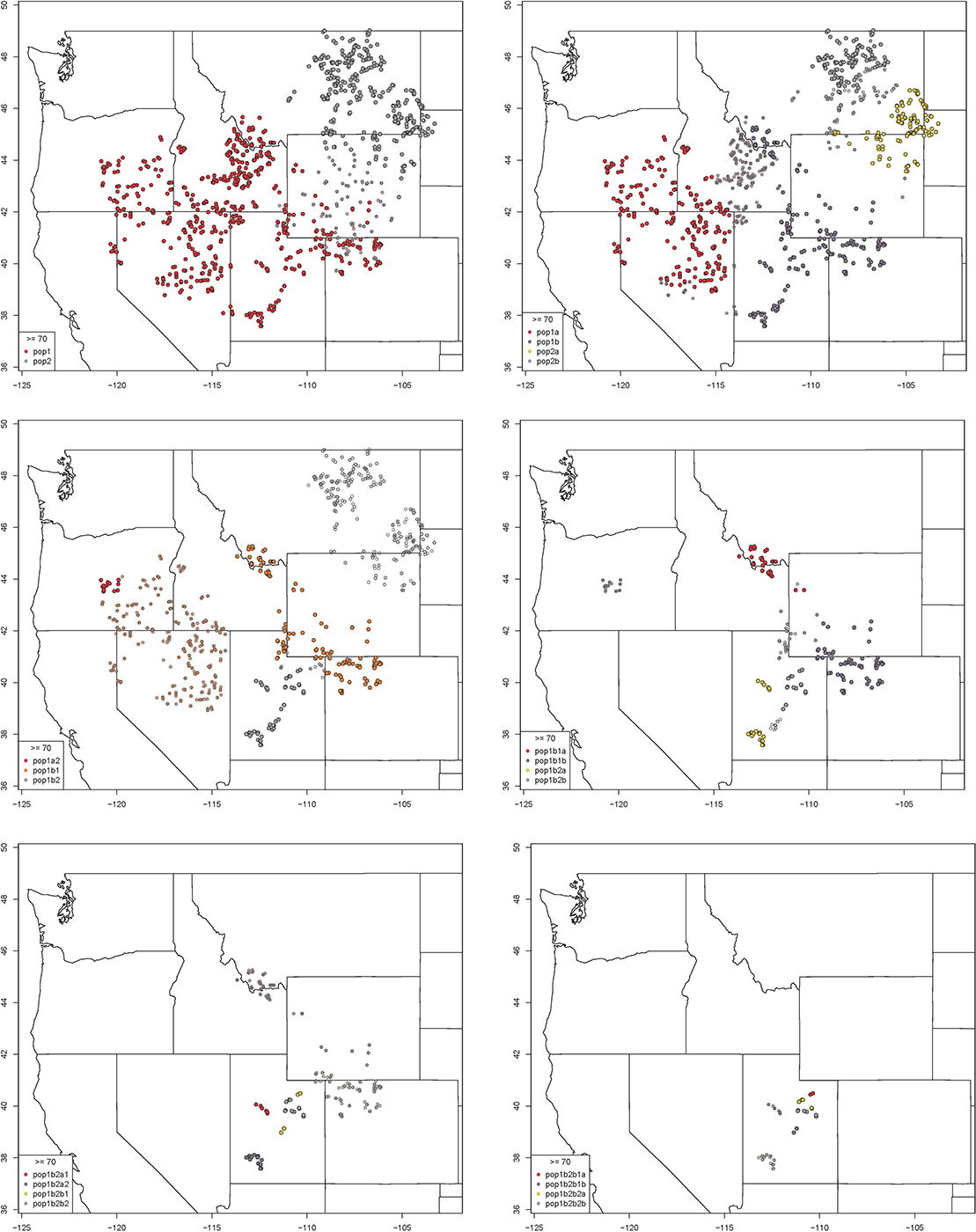

Supplement: S6 Fig — After the first round, two population centers remained ([A] upper left defined by population 1 and 2). After the second round, each population was divided into another two subpopulations ([B] top right defined by pop1a, pop1b, pop2a, and pop2b). Four more rounds of analysis are represented in order by the [C-F]. (JPG) [file pone.0274189.s007.jpg]

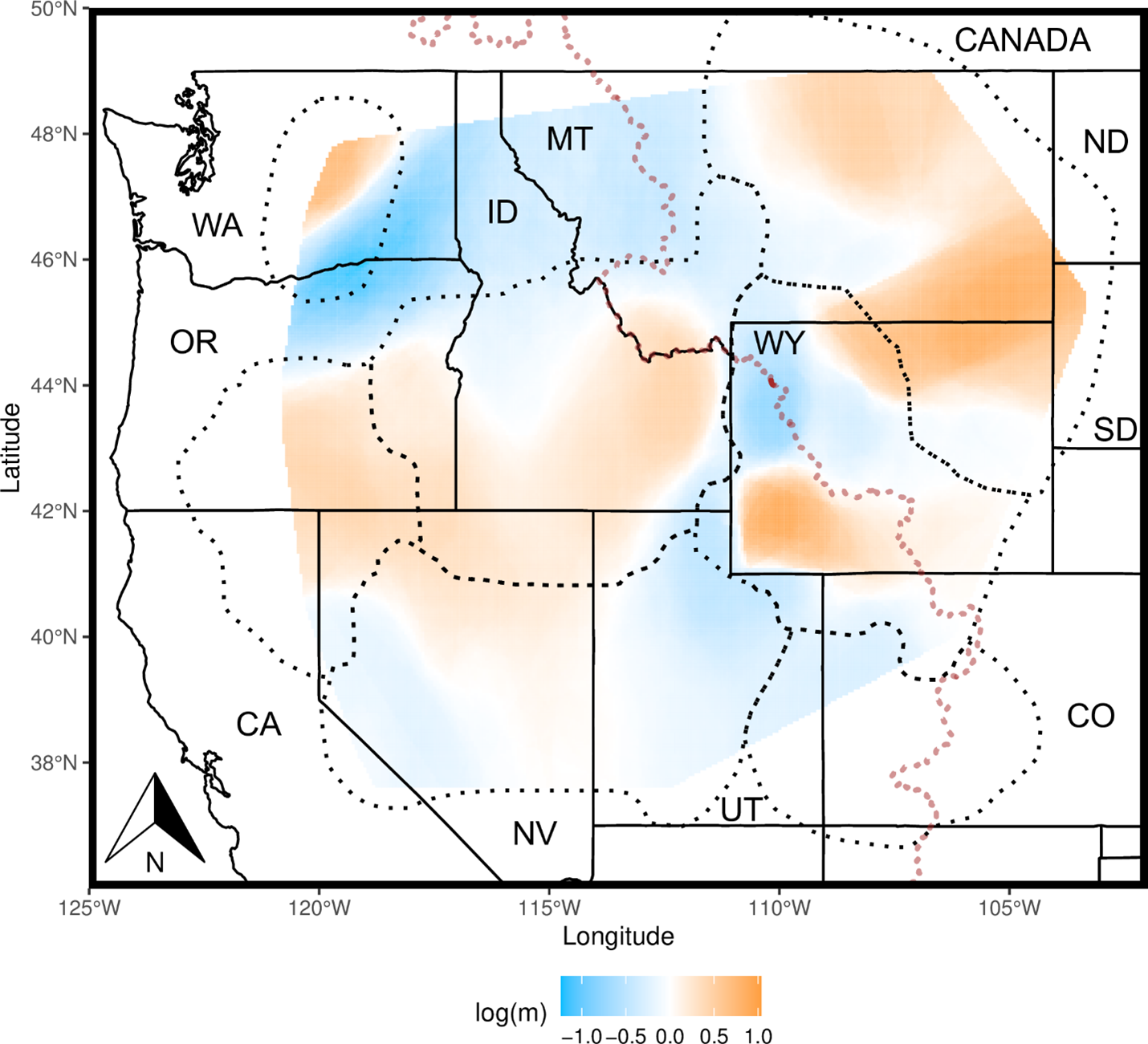

Supplement: S7 Fig — Effective migration rates are shown on a log scale where the zero value indicates the mean effective migration rate, positive values indicate greater than average effective migration (orange) and negative values indicate those less than average (blue). These maps represent the average of models run with a deme count of 500 and 1,000. State names are represented by the following abbreviations California (CA), Colorado (CO), Idaho (ID), Montana (MT), Nevada (NV), North Dakota (ND), Oregon (OR), South Dakota (SD), Utah (UT), Washington (WA), and Wyoming (WY). (TIF) [file pone.0274189.s008.tif]
